# Testing the Level of Social Desirability During Job Interview on White-Collar Profession

**DOI:** 10.3389/fpsyg.2015.01886

**Published:** 2015-12-15

**Authors:** Marek Preiss, Tereza Mejzlíková, Adéla Rudá, David Krámský, Jindra Pitáková

**Affiliations:** ^1^Department of Psychology, University of New York in PraguePrague, Czech Republic; ^2^Department of Psychology, National Institute of Mental HealthKlecany, Czech Republic; ^3^Department of Psychiatry, First Faculty of Medicine, Charles UniversityPrague, Czech Republic; ^4^Police Prezidium, Police of the Czech RepublicPrague, Czech Republic

**Keywords:** justice, moral integrity, balanced inventory of desirable responding, BIDR, desirable responding, personnel selection

## Abstract

Social desirability as a tendency to present oneself in a better light rather than in a truthful manner is common feature presented during job interviews. Previous studies mainly focused on blue-collar professions and therefore authors researched contrary set of white-collar professions in three sub-studies with four different participant groups (legal professions; police officers; controls and university students influenced by scenarios; overall *N* = 636). It was hypothesized that candidates for legal profession would show similar tendency toward social desirability, when compared with controls. Furthermore, police officers were hypothesized to show similar levels of social desirability as legal professions. Lastly, participants in the instruction manipulation condition were hypothesized to show increased levels of social desirability in tender situation as compared to the honest situation. All groups were tested with balanced Inventory of Desirable Responding (BIDR, Paulhus, 1984). Statistical analyses revealed statistically significant differences for both subscales of BIDR when comparing legal professions and control group. Similarly, increased levels of social desirability were detected in police officer candidates as well as in university students in the tender situation compared with students in the honest situation. The overall results indicated that it is typical for white-collar candidates to adapt to the testing situation and it cannot be expected to see different behavior from legal profession candidates as was originally expected.

## Introduction

Social desirability refers to a tendency to respond to self-report items in a way that makes the respondent look good, rather than to respond in an accurate and truthful manner (Holtgraves, 2004). Moreover, it is an important and substantive trait, which relates to a range of responses and behaviors in research, as well as, in the real world (Fleming, 2012). The tendency to influence self-representation and correspondingly results of the interview was found across cultures (Ones et al., 1996; Viswesvaran et al., 2001; Pauls and Crost, 2005; Honkaniemi et al., 2011).

Since blue-collar professions, as well as, professions with low qualifications have been studied excessively (Ones et al., 1993), we chose legal professions requiring high qualifications – in particular judges, public prosecutors and executors, for this study. Based on Plato, a judge should possess four basic characteristics: be able to listen politely, answer wisely, make reasonable considerations and make impartial decisions. Judges and public prosecutors have to be able to work with information, communicate properly and be empathetic, be highly intellectual in order to evaluate all the facts and consequently be able to make decisions, which are fair, objective and independent. For judges and public prosecutors to be able to fulfill all these rules they need to be mature individuals with high moral standards and in a certain sense sometimes be even brave (Vůjtěch et al., 2002).

### Philosophical Perspective

Plato provides an interesting conception of justice, which is a common feature of all three professions represented in the above mentioned sample. Fair soul is composed of three essential components. Plato (Politea) apprehends the restraint (sofrosyné), which is the ability to find the extent of own requests, as the basic prerequisite. The one, who is fair, is then able to give preference to the goodness over his personal desires and needs. The second component, is bravery – masculinity (andreia) in “self-control.” Consequently, a fair soul should, aside from the ability to control his natural needs and desires, stand out among others by being able to control his impulsiveness or anger. He can also dominate in any situation – be master of himself, not allow anger or other passions to take over him and rule him. The last complementary and essential component of the fair soul is understanding – wisdom (sofia). Wisdom is also for Plato, as for Socrates insight because it can be measured – meaning it is able to “reflect” itself as inherently un-finished rather than a virtuoso or competence. In its essence, he paradoxically takes each achievement as “non-rational” or “unfair.”

The fair soul is then primary the one, who Socrates describes (paraphrased by Plato) as “aware of his own ignorance.” Thanks to this “equipment” he is wise – fair human, who is in fact paradoxically the one, who is more than anyone else aware that based on the essence of his fair decisions, they can never be quite fair and also never will be. Thus, the awareness of this discrepancy makes this human a fair one, because a matter of fairness is his life credo and moral commitment, which he is trying to fulfill, even though he knows that he will never be able to do so. Another one of Plato’s large contributions is the unattainable idea of fairness. If the idea of fairness were to be something that one may adopt, and if it were to represent something that one may handle and therefore manipulate, then we could not talk any more about the idea of fairness but about the ideology, which establishes culturally, historically or anthropologically conditioned law.

These philosophical expectations of a judge, public prosecutor or executor characteristics are further supported by the established job descriptions of such positions. For instance when searching for a summary report for judges, public prosecutor and executor the key common feature across all three professions include integrity in sense of honesty and ethical behavior, persistence, active listening, self-control or dependability (O^∗^Net, 2015). Connecting together the work of Plato and current job description, we may expect to see all these characteristics in job candidates pursuing these positions. Therefore, it is reasonable to expect low levels of contradictory behaviors, such as tendencies to respond in a socially desirable manner instead of responding in an honest way.

### Social Desirability in Testing Situations

The majority of research articles, studying the impact of response styles on results of psychological assessments administered during evaluations, are based on instruction manipulations that are directed by researchers. Studies focusing on a behaviors in real situations are far less frequent. While according to some findings, social desirability scale scores may have real-life impact and be expected to be predictive of individual differences in job performance (Viswesvaran et al., 2001). These findings were contradictory with Ones et al. (1996), who found for managerial professions very low corrected correlation between social desirability and overall job performance. In the discussion concerning impression management responding style on different professions, mentioned new meta-analysis by Viswesvaran et al. (2001) was published. In this meta-analysis authors concluded that in profession, such as management, where interpersonal interactions are essential, impression management scales are weak in predicting job performance. For the purpose of presented study it might be hypothesized that some factors measured by these authors (e.g., management disagreements, building relationships, effective speaking, acting with integrity, analyzing issues) might be similar to legal professions, but no relevant specifically oriented studies in real situations concerning this profession were found.

Substantial attention is paid toward the intentional influencing (faking) of results in personality tests, which are often used during job interviews. Research results showed that all Big Five variables may be easily influenced (Viswesvaran and Ones, 1999). Birkeland et al. (2006) compared the results of 33 studies in their meta-analysis, using personality tests based on the Big Five theory, where participants consisted of real job applicants and those, who were not seeking a job. Independently of the job type, job applicants scored significantly higher on scales of Extraversion, Emotion stability, Conscientiousness and Openness toward new experiences when compared to those, who did not seek a job. In case of particular occupations (such as salesmen), differences found in the psychological variables that were perceived as related to the given job position from the potential employer’s perspective, were even more distinct. The differences found under real job interview conditions were smaller, as opposed to studies, which used the results of experimental comparisons with instruction manipulations directed by the researcher (faking good, faking bad).

Furthermore, job applicants use various kinds of strategies to achieve their desired goal of being employed, such as self-promotion, when the applicant presents that he or she possesses desired qualities of the given job. Other strategies encounter for example ingratiation, to serve as an effort to awaken sympathy and feelings of attraction, or to serve as defense tactics with an effort to protect and reform the candidate’s image. There is a model designed for understanding patterns of intentionally influencing results of job interviews results by applicants (Levashina and Campion, 2006). This model proposes 19 measurable ways of observing applicant’s intentional influencing of results during the interview (Levashina and Campion, 2006). Authors proceeded from a number of assumptions, such as job applicants with low integrity will have higher desire to intentionally influence results of the assessment. They argue that intentional influencing of results during the interview is caused by the *capacity* (e.g., of verbal abilities, social abilities, knowledge about the position), *effort* (e.g., personal characteristics, integrity, low probability that the applicant may get caught) and *the opportunity for intentional influencing* (e.g., interview method, respondent’s interests). Additionally, these authors claim that structured interviews do not allow for intentional influencing of results as much when compared with unstructured interviews.

Influencing results of a selection procedure by applicants is relatively frequent, despite the fact that human resource specialists’, as well as, the personnel psychologists’ ideal would be a person, showing stability and providing undistorted information about him or herself. The pattern of responses are measured using the Paulhaus’ Balanced Inventory of Desirable Responding (BIDR; Paulhus, 1984). The BIDR method is often used in research as an additional scale of personality testing (Booth-Kewley et al., 1992; Porter et al., 2000; Honkaniemi et al., 2011). For example, a study done by Finish researchers (Honkaniemi et al., 2011) investigated school applicants and their evaluations of selection procedures and post-completion questionnaires concerning the level of social desirability. Respondents were asked to express their opinions about the process of the interview and to further fill out BIDR questionnaire. The results of the study showed that respondents with more positive evaluations of the selection process had higher scores on the Impression management scale but not on the Self-deception scale.

### Focus of the Present Study

In a case of justice applicants, the selection process altogether consists of five stages (Vrcha, 2006). Therefore, it is lengthy and takes several months. The goal of the psychological assessment is to evaluate characteristics and other personal predispositions of the applicant. This is ensured by the chairman of the regional court, while the assessment is carried out at specialized psychological departments chosen by the Ministry of Justice. The psychological assessment is the last stage of the entire selection process. In some types of justice tenders, the psychological assessment is just one of many stages in the selection procedure, however, for most applicants, it is the last obstacle to overcome and reach their goal.

The present study explored, whether people, who attended university and spent large amount of time preparing for their profession in justice (connected with fairness, deliberation, and wisdom), have a tendency toward distorted style of responding during the last stage of the selection process. In another words, whether or not applicants will have a tendency toward unreal and desirable self-representation.

It is assumed that the responding style of these people should not include increased tendency of self-deception or to be deceptive toward others. It is further assumed that future judges, public prosecutors, and executors will possess a tendency toward open and sincere responding styles in their everyday lives, as well as, during the tender selection process. In order to test this hypothesis, the authors performed three studies. During the first study, authors compared the tendency toward socially desirable responding between legal professions and a control group. It was hypothesized that legal profession candidates would show similar tendency toward social desirability as their matched controls (H1). This hypothesis was based on the nature of the legal profession, which includes high integrity, morality and ethical behavior, as described in the previous studies above (Vůjtěch et al., 2002), and Plato’s (2014) work and summary reports of these professions (O^∗^Net, 2015). In the second study, authors chose similar but a different profession (police officer candidates), to see whether the tendency toward social desirability may differ across similar professions. Given the nature of the profession that police officers serve, it was hypothesized that both legal profession and police officer candidates would show similar levels of social desirability (H2). This hypothesis was based on the similarities of legal obligations and moral standards in these professions. Further, this hypothesis was based on the identical condition of the tender situation, which may play a crucial role in the assessment and the condition itself, while it may also be a reason for increased levels of social desirability. Finally, in the third study, authors compared the tendency toward social desirability with people from general population using two scenarios. One scenario asked participants to answer honestly and openly with no external offer present, whereas the second scenario asked participants to answer as if there was an external offer in a form of a job offer. Thus, in the third study, authors focused on the influence of instruction and participant’s tendency toward socially desirable responding. It was hypothesized that participants in the instruction manipulation condition would show increased levels of social desirability in tender situation as compared to the honest situation (H3). This hypothesis was based on the assumption that regardless of the profession instruction inducing tender situation would be crucial for the tendency toward social desirability.

## Materials and Methods

In the following studies we tested several different groups, however, as the distribution into the groups lack essential features of randomization, the design of the presented study was quasi-experimental. Since the groups were not matched properly the specific type of quasi-experiment we have used was non-equivalent comparison groups, as the groups included participants of various age and level of education. For the statistical analyses below we have used mixed design. The mixed design included between-subject comparisons, where we compared how groups differed from each other, and within-subject comparisons, where we looked at the differences within the groups (e.g., gender differences). The instrument used in this quantitative research was the Balanced Inventory of Desirable Responding Czech version, which was translated and published with the permission of Paulhus (1984). The psychometric results of the Czech version of this inventory were already published (Preiss and Mačudová, 2013) with satisfactory internal consistency – the level of Cronbach’s alpha for the Self-deception scale was 0.70 and for the Impression management scale 0.80.

### Self-Deception Scale (SDE)

The first 20 questions (1–20) of the BIDR questionnaire measure self-deception (sometimes also referred to as “ego defense”), which is represented with questions such as “it would be difficult for me to get rid of some of my bad habits” or “I always know, why I like certain things.” The self-deception scale represents honest responding style, which is on the other hand often disrupted by the unconscious tendency of improving self-image. The scale scores could be also interpreted as faking good or faking bad.

### Impression Management Scale (IM)

The second 20 questions (21–40) of the BIDR questionnaire measure impression management, which is represented with questions such as “when I overheard somebody else’s private conversation, I tried not to listen” or “sometimes I drive faster than what is allowed.” The scale was established in order to detect respondent’s effort to behave in a socially desirable manner and is labeled as a measure of “grace exaggeration.” The scale is also sometimes described as a lie or fake scale and could be accented in the desired or undesired direction. In comparison with the previous scale, this scale is valued as more important from the perspective of possible influencing of the questionnaire scores.

### Study 1 – Legal Professions

The procedure of the psychological assessment followed criteria for candidate evaluations in correspondence with the regulations issued by the Ministry of Justice and the appropriate amended legal code. Assessments of future judges, public prosecutors and executors were done individually and in correspondence to the contract with the Ministry of Justice. The assessment consisted of anamnestic interview, observation, and several other paper-pencil based methods. The BIDR was also part of the assessment. Participants from the original validation study (Preiss and Mačudová, 2013), who voluntarily and without any remuneration participated in the research and filled out questionnaires under non-testing condition, were matched to the experimental group based on age and gender after the assessment of all experimental group participants (future judges, public prosecutors and executors).

#### Participants

##### Experimental group

The experimental group consisted of 58 participants, where 27 were males and 31 females. The inclusive criteria were in correspondence with above mentioned regulations issued by the Ministry of Justice. Thus participants in this group came on the recommendation of the given ministry and were in the last phase of the assessment to become a judge, public prosecutor or executor. Correspondingly, exclusive criteria was an inability to succeed in the previous stages of the assessment (e.g., insufficient qualification, failure in legal code exam etc.). The mean age in this group was 29.4 years (*SD* = 5.4). In terms of education, all participants obtained a university degree. The experimental group was in a real testing situation and results of the assessment had a practical impact on the observed candidates in terms of the multilevel selection procedure.

##### Control group

The control group consisted of 56 participants, where 27 were males and 29 females. Participants, who showed an interest to complete the questionnaire, worked on volunteer basis and lacked any psychological condition were included into the study. Participants, who positively answered at least one of the following questions (Have you ever been treated with a psychological condition? Has your biological father, mother, brother or sister been ever treated with a psychological condition? Have ever been unconscious for longer than 5 min? Have you ever had problems with overdose or addiction to drugs (e.g., marihuana, methamphetamine etc.)? Have you ever overdosed medications? Are you treated for any medical conditions? Are you currently using medication that could influence your mood or performance? Have you ever had an epileptic seizure? Have you ever had problems with overdose or addiction to alcohol?) were excluded from the study (see original validation article, Preiss and Mačudová, 2013). The goal of this initial screening was to include only relatively healthy participants from general population. The mean age of this group was 32.0 years (*SD* = 14.3). In terms of education, 86% of participants obtained a university degree or graduated from high school, the rest of participants had a lower level education. Participants were chosen from the original validation study (Preiss and Mačudová, 2013). The individuals from this group were assigned based on their age, sex, and educational level to the participants from the experimental group.

#### Procedure

The BIDR was administered as a part of the psychological assessment together with other questionnaires. The questionnaire was administered with the instruction to rate each statement based on how much the participant agreed with it, using the appropriate number from the scale below. Respondents rated their answers on a Likert type scale ranging from 1 to 7, where 1 represented “not true at all” and 7 “absolutely true.” For interpretation purposes, scores on each of the scales were added individually, as well as, together for the overall scale score. The increased overall BIDR score could be interpreted in terms of a general tendency toward socially desirable responding, deception, and self-deception.

### Study 2 – Police Officers

#### Participants and Procedure

Data for the second study were obtained again in real testing situation from job candidates pursuing a police officer position (*N* = 360) at the police department of the Czech Republic. All participants included in this group came based on the recommendation of the Czech Ministry of the Interior, where psychological assessment was a mandatory part of the assessment. Correspondingly, exclusive criteria included an inability to succeed in the previous parts of the assessment (e.g., insufficient qualification). BIDR was administered as a part of the evaluation procedure during a standard and complex psychological assessment at individual psychological departments across Czech Republic. The average age of participants was 26.3 years (*SD* = 7.2; 18–56 years, 87% were males). With regard to education, most participants obtained some kind of secondary degree (78%), the rest of the sample held a university degree.

### Study 3 – Scenarios with General Population

#### Participants and Procedure

Data of the BIDR questionnaire for the third study were collected from university students of an external educational program as a part of their ethics course. Participants, who showed an interest to complete the questionnaire, worked on volunteer basis and were presented in the classroom during the entire class hour were included into the study. Correspondingly, participants, who did not want to participate in the study were excluded. The data for the honest condition of the BIDR questionnaire with the instruction to answer “in a way you usually are” was gathered from 162 participants. The mean age of this group was 32.5 (*SD* = 9.5, 19–68, 48% were males). Subsequently, 127 participants from the same group with the mean age of 32.0 (*SD* = 8.4, 20–51, 51% were males) filled out the BIDR questionnaire with the instruction to imagine as if they were attending a job interview for a desired position (tender condition scenario). Both collections were done during the same class hour.

### Ethical Standards

The presented study was approved by the ethical committee of the Prague Psychiatric Center, Ústavní 91, Prague 8-Bohnice, 18103 and have therefore been performed in accordance with the ethical standards laid down by the Declaration of Helsinki in 1964. Prior to the inclusion into the study each participant gave a consent agreeing with the assessment. Moreover all data were handled in a confidential manner and subjects were kept anonymous.

## Results

### Study 1 – Legal Professions

At first, data were analyzed using Shapiro–Wilk test verifying normal distribution. Results of this analysis revealed normal distribution (*W* = 0.983, *p* = 0.159) and therefore, parametric tests were used for further analyses. **Table 1** shows the results of BIDR-CZ for candidates in the legal professions (testing condition) and their matched control group (non-testing condition). An independent sample *t*-test revealed that self-deception was greater in legal profession candidates as compared to their matched controls (*t* = 6.704, *df* = 110, *p* < 0.001). Similar results were also observed for impression management, where legal profession candidates scored significantly higher than their matched controls (*t* = 6.683, *df* = 110, *p* < 0.001). Hypothesis 1, stating that candidates for legal profession would show similar tendency toward social desirability when compared with controls, was rejected.

**Table 1:** The results of BIDR-CZ for candidates of legal professions and matched control group.

	*N*	Self-deception scale (SDE)	Impression management scale (IM)
Legal professions candidates	58	98 (*SD* = 13; 69–122)	96 (*SD* = 22; 42–130)
Control group for legal professions candidates	56	82 (*SD* = 13; 54–111)	71 (*SD* = 17; 34–111)


**Table shows mean values, SDs, minimal and maximal scores.**

### Study 2 – Police Officers

**Table 2** shows the average BIDR scores of candidates pursuing a police officer position or legal profession position. In this study both groups were in the testing condition. Prior to the statistical analysis normality of distribution was verified using Shapiro–Wilk, which indicated that the data were not normally distributed (*W* = 0.991, *p* = 0.01). Therefore, non-parametric tests were utilized. A Mann Whitney U indicated that the tendency toward self-deception was the same for legal professions and for police officer candidates (*U* = 9634.5, *p* = 0.346). Similar results were shown when looking at the impression management, where these two groups also did not differ (*U* = 9529, *p* = 0.286). Hypothesis 2, stating that police officers will show similar levels of social desirability as legal professions, was supported.

**Table 2:** The results of BIDR-CZ for candidates of legal professions and police officers.

	*N*	Self-deception scale (SDE)	Impression management scale (IM)
Legal professions candidates	58	98 (*SD* = 13; 69–122)	96 (*SD* = 22; 42–130)
Police officer candidates	360	96 (*SD* = 15; 53–132)	94 (*SD* = 19; 44–138)


**Table shows mean values, SDs, minimal and maximal scores.**

### Study 3 – Scenarios with General Population

**Table 3** shows results obtained from university students for the honest condition and the tender condition. A sign test indicated that when students were asked to answer honestly, self-deception scores were significantly lower than when they were asked to answer as if attending a job interview (*Z* = 6.49, *p* < 0.001). Similar results were obtained for the impression management scales, where again students in the honest condition showed significantly lower scores as compared to the tender condition (*Z* = 6.24, *p* < 0.001). Hypothesis 3, stating that participants in the instruction manipulation condition would show increased levels of social desirability in a tender situation as compared to a honest situation, was supported.

**Table 3:** The results of BIDR-CZ for candidates of legal professions and their matched control group, police officer candidates, honest and tender condition scenarios.

	*N*	Self-deception scale (SDE)	Impression management scale (IM)	Differences between samples for SDE and IM
Legal professions candidates (Sample 1)	58	98 (*SD* = 13; 69–122)	96 (*SD* = 22; 42–130)	Sample 1 vs. sample 2 and 4^∗∗∗^
Control group for legal professions candidates (Sample 2)	56	82 (*SD* = 13; 54–111)	71 (*SD* = 17; 34–111)	Sample 2 vs. sample 1, 3, 4^∗∗∗^
Police officer candidates (Sample 3)	360	96 (*SD* = 15; 53–132)	94 (*SD* = 19; 44–138)	Sample 3 vs. sample 2 and 4^∗∗∗^
Honest condition (Sample 4)	162	84 (*SD* = 16; 0–123)	80 (*SD* = 18; 0–133)	Sample 4 vs. sample 1, 3, 5^∗∗∗^
Tender condition (Sample 5)	127	99 (SD = 21; 0–144)	99 (*SD* = 24; 0–140)	Sample 5 vs. sample 2 and 4^∗∗∗^


**Table shows mean values, SDs, minimal and maximal scores. ANOVA, *post hoc* analysis, Tukey HSD test. Significant at the level ^∗∗∗^*p* < 0.001.**

### Differences Among All Samples

Statistical analyses of scale scores and demographics revealed that correlation (Pearson) was detected in five groups between SDE and age ranging from *r* = -0.11 to *r* = 0.21. Nevertheless, a significant correlation between self-deception and age was found only among the police officers group (*r* = 0.21, *p* < 0.001). In terms of impression management and age, Pearson correlation ranged from *r* = -0.18 to 0.16. Nonetheless, neither correlation among the groups was found to be significant.

Among the legal profession candidates, significant differences between SDE scores and gender were found. In particular, one-sample *t*-test indicated significantly higher SDE scores for men than for women (*t* = -3.06, *df* = 136, *p* = 0.003). Similar results were found in the group of police officer candidates, where men showed significantly higher scores in SDE (*U* = 3400, *p* < 0.001), as well as, in IM scores (*U* = 3824.5, *p* < 0.001) when compared with women candidates. Neither the group of university students nor the control group showed significant differences between genders.

For the SDE scores, *post hoc* analysis (ANOVA, Tukey HSD test) revealed that legal profession candidates scored significantly higher than their control group (*p* < 0.001) and students in honest situation scenario (*p* < 0.001), but there was no difference found among legal professions and police officers (*p* = 0.874) or students in the tender situation scenario (*p* = 1.00). Similarly, this analysis showed no difference between police officers and students in the tender situation scenario (*p* = 0.473), but showed higher SDE scores when compared with control group (*p* < 0.001) and students in the honest situation scenario (*p* < 0.001). Furthermore, analyses indicated that SDE scores did not differ significantly between control group and students in the honest situation (*p* = 0.867).

For the IM scores, *post hoc* analysis (ANOVA, Tukey HSD test) revealed that legal profession candidates scored significantly higher than their control group (*p* < 0.001) and students in the honest situation scenario (*p* < 0.001), but there was no difference found among legal profession candidates and police office candidates (*p* = 0.965) or students in tender situation scenario (*p* = 0.728). Contradictory, students in the tender situation showed significantly higher scores than police officer candidates (*p* = 0.04). Furthermore, analyses revealed that IM scores were significantly lower for control group than for students in the honest situation scenario (*p* = 0.019).

## Discussion

Due to the fact that participants were not assigned to given groups randomly, the design of this quantitative research was quasi-experimental. The dependent variable was the score obtained on the BIDR-CZ questionnaire, while the independent variable was the condition in which participants were in, being either the condition with an external offer (job interview) or without an external offer (control or honest group). As these groups were not equivalent the specific type of quasi-experiment used was non-equivalent comparison groups design. Participants in each group differed in sociodemographic variables such as age or level of education. Given the nature of quasi-experiment, researchers were not able to draw causal relationships. From the statistical perspective we have used the mixed design comprising of between-subject and within-subject comparisons. The between-subject comparisons allowed for discovering differences between individual groups and their levels of social desirability. The within-subject comparisons allowed for searching differences among individuals within the same group undergoing the same conditions, so for example the analyses looked at a tendency toward social desirability between men and women.

The statistical analyses revealed that legal profession candidates showed statistically higher tendency toward social desirability on both scales (<0.001) when compared with control group, thus rejecting H1. The hypothesis was originally based on the nature of the legal profession as described in previous studies (Vůjtěch et al., 2002), and Plato’s (2014) work and summary reports (O^∗^Net, 2015). However, as results showed, it is possible that the influence and pressure associated with tender situation may play more significant role and thus suppress the personal attributes related to integrity or honesty. This assumption was further supported by the second study evaluating the tendency toward socially desirable responding in police officer candidates. Here, statistical analyses again revealed higher social desirability scores in group of police officer candidates when compared with control group (*p* < 0.001), but did not reveal significant differences between police officer candidates and legal profession candidates (SDE- *p* = 0.346; IM- *p* = 0.286, supporting H2). Both groups participated in job interview and were aware that the results of the overall assessment would have an impact on the overall results of the job interview. Further to examine the influence of tender situation on responding style in a same group of participants, by just using instruction manipulation, Study 3 was conducted. In this study, it was hypothesized that regardless of the profession, instruction inducing tender situation would impact the tendency toward social desirability. This hypothesis was supported when students were asked to answer as if they were attending a job interview, where they would like to succeed. Their scores were significantly higher when compared with the condition when they were supposed to answer the items honestly based on how they are usually (*p* < 0.001). Thus, these results imply that regardless of the given profession, the situation of a job interview is so specific and influential that differences across professions may be diminished. However, for this conclusion to be true, we would certainly need comparisons across variety of professions, which we do not have.

Within the statistical analyses, there were two anomalies, which were not presumed prior to the study. First, *post hoc* analysis of the IM scores showed that students in tender situation condition showed significantly higher scores than police officer candidates (*p* = 0.04) but they did not differ from legal profession candidates (*p* = 0.728). It this case it is important to notice that the significance is only marginal and students were not in a real situation, thus their socially desirable behavior may have been exaggerated. Second, *post hoc* analysis of the IM scores revealed significantly lower scores in the control group as compared to the students in the honest situation condition (*p* = 0.04). This result may have been influenced by the environment, in which questionnaires were administered to both groups. Despite the fact that students were asked to answer honestly, the questionnaire was still administered to them during an ethics course and therefore, this may have had an influence on their level of social desirability.

Overall, the results of this study showed significant differences in tendency toward socially desirable responding style. If there was an explicit offer, whether (legal profession and police officers candidates) or not (simulated as in tender condition in Study 3) it was real, all three groups showed increased tendency toward social desirability. Legal profession group did not differentiate itself from the other two groups in a tendency toward socially desirable responding style. The average score of all three groups ranged between 96 – 99 points for SDE and 94 – 99 points for IM. Contrary, the level of social desirability in both control groups was significantly lower (SDE = 82 – 84 points, IM = 71 – 80 points).

The increased tendency toward socially desirable style of responding is supported by the comparisons of the results with our previous study validating BIDR on the Czech population (Preiss and Mačudová, 2013). For legal professions, in comparison with the original participants of the control group (Preiss and Mačudová, 2013), the experimental group reached 93th percentile on the SDE scale and 76th percentile on the IM scale. For the police officer group, in comparison with the original participant control group (Preiss and Mačudová, 2013), the experimental group reached 90th percentile on the SDE scale and 74th percentile on the IM scale. In comparison with the original participant control group (Preiss and Mačudová, 2013), the group in tender condition scenario reached 90th percentile on the SDE scale and 94th percentile on the IM scale.

The advantage for this study consisted of the real testing environment and the ability to include participants from professions with an extraordinary social importance, such as judges, legal professions, and police officers. The legal professions group consisted of participants that were highly educated as well as used to different types of testing situations, and they had experiences with various kinds of examinations that influenced their career (such as BAR exam; postgraduate studies etc.). Moreover, the detection of faking in the presented groups is another advantage of this study, showing the strengths of the BIDR questionnaire. Previous studies showed that it is more difficult to detect faking in groups of highly educated and intelligent people. The intelligence relates to the consciousness of influencing results and the ability to sense situational challenges (Pauls and Crost, 2005; Levashina et al., 2009).

### Interpretations of BIDR Scores

The key question here is the interpretation of BIDR results. One may speculate about the possibility of interpretations in a positive, as well as, negative aspects as seen below:

(1) The extent of the overall assessment validity, while using a variety of methods (BIDR-CZ within the norm showed sufficient assessment validity), and willingness to provide undistorted answers.
(2) General tendency toward a specific responding style, desirable self-representation, overestimation of own abilities, ignorance of self or possible pretending, and in extreme cases even faking.
(3) The extent of exaggeration of “virtue” (IM scale) as a more of a trait-like component, a desire to leave a positive impression.
(4) The extent of SDE scale, as honest, yet exaggerated characteristic of self, more of a trait-like component.
(5) Positive, adaptive abilities (in a case of score elevations).

#### Negative Interpretation of High BIDR Scores

Accurate self-knowledge is defined as knowledge of one’s personality traits as they are exhibited in behavior (Vogt and Colvin, 2005), and it is expected to be a part of mental health. Self-enhancement bias seems to be related to narcissism (Robins and Beer, 2001) and self-enhancing beliefs may be adaptive in short term but not in the long term. Longitudinal study done by Paulhus (1984) looked at seven group meetings and discovered that during the first meeting self-enhancers made positive impressions on the rest of the group. Contrary, the group rated self-enhancers negatively after 7 weeks and gave discrepant self-evaluations with self-enhancer’s evaluations. In another longitudinal study, which was based on observer’s ratings performed 5 years before and 5 years after the assessment of self-enhancement, self-enhancement was associated with poor social skills and psychological maladjustment (Colvin et al., 1995). In Colvin and Griffo’s (as cited in Chang, 2008) review concerning self-enhancement, authors stated that self-enhancement is associated with general maladjustment and is primarily focused on a behavioral syndrome similar to narcissistic personality disorder.

#### Positive Interpretation of High BIDR Scores

The first four points displayed BIDR as a tool for discovering problematic responding style (distorted answers). Furthermore, some authors (Pauls and Crost, 2005; Honkaniemi et al., 2011) even talked about direct faking or lying. Nevertheless, the fifth point may present social desirability as neutral or even positive attribute within the job selection process or in the context of one’s work performance. Therefore, it is possible to argue (Pauls and Crost, 2005) that conscious influencing does not have to be necessarily perceived as a risk for the validity of the finding, but more as positive, adaptive, and probable predictive characteristic, which should be studied more on its own. Rosse et al. (1998) stated that individuals, who are able to influence their responses in socially desirable manner are more aware of social norms, hence competent of a better performance. Based on study done by Jansen et al. (2012), self-representation in a selection situation highly corresponded with employer’s expectations, whether the candidate tried to consciously or unconsciously fulfill the employer’s expectations. When applicants (in this case applicants were university absolvents from Switzerland searching for a job) did what was expected of them, in most cases we should not morally judge them for that (Jansen et al., 2012). Similar thoughts were expressed also by Ruch and Ruch (1967) or Levashina and Campion (2006), who believed that impression management, could be considered as a valid predictor of future work performance, such as in a case of salesmen or spokesmen. Some authors (Jansen et al., 2012) argued that self-representation with the effort to accept requirements of the other side, do not represent a desire to behave in a socially desirable way; instead, it represents the actual behavior. However, job applicants should know that strategic self-representation is acceptable during job interview situation and that they need to be able to “sell” themselves.

### Contradictory Studies

Essential information about social desirability and work performance were provided in a discussion of a study done by Christiansen et al. (2010). These authors (Christiansen et al., 2010) claimed that social desirability does not have a relationship with work performance. They cited an extensive study (Ones et al., 1996), which found a 0.01 correlation with the overall work performance among a variety of participants’ groups. These authors (Ones et al., 1996) also did not find any predictive abilities of socially desirable scales toward a school performance, test performance, counterproductive work behavior and work performance. Moreover, they assumed that the influence of social desirability is exaggerated in the personnel psychology and that it reflected individual differences between emotional stability and consciousness. This study was further criticized by Rosse et al. (1998) who emphasized that Ones and colleagues did not use Paulhaus’s two factor structure of socially desirable responding in their study.

Another study that contradicted the importance of social desirability (Viswesvaran et al., 2001) and compared impression management and manager’s performance across more than 20 000 participants found that impression management did not have more marked influence (*r* = 0.04) on manager’s work success. In a group of more than 800 managers, the authors found a correlation between supervisor’s evaluation and social desirability of the evaluating employee ranging from -0.06 to 0.07, which again was not significant. At the same time, these authors emphasized that the study was performed only on one professional group (managers). From the long-term perspective, marked tendency to behave in a deceptive way could be counterproductive and from the organizational side perceived as inappropriate (Jansen et al., 2012).

### Implications of this Study

It is possible to agree with at least the assumption that the work of judges, public prosecutors and executors is vastly different from that of sales persons or managers. If Socrates’s remarks about judges are true – stating that judge should possess the following characteristics – be able to listen politely, answer wisely, make reasonable considerations, make impartial decisions – this is quite a different personality profile when compared to managers where flexibility, ability to make quick decisions even in a stressful situation, and communication of high quality is expected – these are also preferable characteristics of public prosecutor or executor (O^∗^Net, 2015). Similar thoughts were also expressed by Rosse et al. (1998), who argued that social desirability could be positive for certain types of jobs. Good examples are jobs requiring more shallow human contact or one-time contacts with customers. High levels of social desirability could be disadvantageous or even dysfunctional in other types of professions, such as for auditors or professions requiring long-term cooperation. Judicial professions, public prosecutors, and executors are more in correspondence with the second alternative. The stylization of answers is clearly not just a question of social desirability but in extreme cases, it is instead the question of integrity (Rosse et al., 1998).

Prior to this study, we favored in compliance with these opinions the interpretation of the elevation of a level of social desirability found using the BIDR method, as negative, considering the expected moral integrity connected with the justice profession on a position of a judge, public prosecutor and executor. However, the presented study changes our assumptions. It seems that despite the general expectations of personal stability, integrity and wisdom associated with legal professions, this group of participants is just as susceptible to social desirability as any other profession (such as police officers) or other participants in a situation of an external offer (tender situation scenario). Results of our study suggest that social desirability does not have to dependent upon a profession and we may assume that it is a common feature in situations promising a reinforcement or a gain of any sort. We may therefore conclude that the essential part is the situation and the offer in itself – or the reinforcement/gain that comes out of it.

### Limitations

The basic limitation of our study was that the examined groups had different number of participants and that the testing situation came out of specific conditions in just one country. During the administration of the BIDR, as well as, during the administration of other methods for psychological assessment, participants were not warned about the need to fill out BIDR, as well as, other methods in open and truthful manner. The contrary instruction could have influenced the study results. In a clinical field, during administrations of effort evaluations (e.g., compensations evaluations, lawsuit), participants are instructed at the beginning to answer openly and truthfully. Moreover, they are warned that methods allow for the answer validity assessment. In the context of the experimental group assessment this was not done.

### Future Studies

In correspondence with limitations of the presented study, future studies should focus on a variety of social desirability measures and administer them among the assessment batteries. Furthermore, the instructions should include the possibility for the answer validity assessment and encourage open and truthful answers. Authors also believe that future studies should still keep their focus on these groups of participants aside from the blue-collar professions since these data are not available, and that they should possibly broaden the field with cross-cultural data.

## Author Contributions

MP is the first and main author of this article, he worked on the conception and the design of the research, data collection and further analyzed and interpreted the data results. He also drafted the original article and contributed to further revisions. TM as the second author worked on the conception of the study and data interpretation and also on the draft and further revisions of the final article. AR worked on the design of the study and also collected all the data for the police officers sample, further she participated on the draft of the proposed article. DK cooperated on the design of the study, collected some of the data for honest and tender scenarios and participated on the draft of the proposed article. JP helped with the interpretation of the data and participated on the draft of the article and necessary corrections.

## Conflict of Interest Statement

The authors declare that the research was conducted in the absence of any commercial or financial relationships that could be construed as a potential conflict of interest.

## References

[B1] Birkeland S. A. Manson T. M. Kisamore J. L. Brannick M. T. Smith M. A. (2006). A meta-analytic investigation of job applicant faking on personality measures. *Int. J. Sel. Assess.* 14 317–335. 10.1111/j.1468-2389.2006.00354.x

[B2] Booth-Kewley S. Rosenfeld P. Edwards J. E. (1992). Impression management and self-deceptive enhancement among Hispanic and non-Hispanic White navy recruits. *J. Soc. Psychol.* 132 323–329. 10.1080/00224545.1992.9924707

[B3] Chang E. C. (2008). *Self-Criticism and Self-Enhancement: Theory, Research, and Clinical Implications.* Washington, DC: American Psychological Association, xvi, 291 123–140.

[B4] Christiansen N. D. Rozek R. F. Burns G. (2010). Effects of social desirability scores on hiring judgments. *J. Pers. Psychol.* 9 27–39. 10.1027/1866-5888/a000003

[B5] Colvin C. R. Block J. Funder D. C. (1995). Overly positive self-evaluations and personality: negative implications for mental health. *J. Pers. Soc. Psychol.* 68 1152–1162. 10.1037/0022-3514.68.6.1152 7608859

[B6] Fleming P. (2012). Social desirability, not what it seems: a review of the implications for self-reports. *Int. J. Educ. Psychol. Assess.* 11 3–22.

[B7] Holtgraves T. (2004). Social desirability and self-reports: testing models of socially desirable responding. *Pers. Soc. Psychol. Bull.* 30 161–172. 10.1177/0146167203259930 15030631

[B8] Honkaniemi L. Tolvanen A. Feldt T. (2011). Applicant reactions and faking in real-life personnel selection. *Scand. J. Psychol.* 52 376–381. 10.1111/j.1467-9450.2011.00892.x 21752026

[B9] Jansen A. Cornelius J. K. Stadelmann E. H. Kleinmann M. (2012). Applicants’ self-presentational behavior. What do recruiters expect and what do they get? *J. Pers. Psychol.* 11 77–85. 10.1027/1866-5888/a000046

[B10] Levashina J. Campion M. A. (2006). A model of faking likelihood in the employment interview. *Int. J. Sel. Assess.* 14 299–16. 10.1111/j.1468-2389.2006.00353.x

[B11] Levashina J. Morgeson F. P. Campion M. A. (2009). They don’t do it often, but they do it well: exploring the relationship between applicant mental abilities and faking. *Int. J. Sel. Assess.* 17 271–281. 10.1111/j.1468-2389.2009.00469.x

[B12] O^∗^Net (2015). *Summary Report for Occupations.* Available at: http://www.onetonline.org/

[B13] Ones D. S. Viswesvaran C. H. Schmidt F. L. (1993). Comprehensive meta-analysis of integrity test validities: findings and implications for personnel selection and theories of job performance. *J. Appl. Psychol.* 78 679–703. 10.1037/0021-9010.78.4.679

[B14] Ones D. S. Viswesvaran C. H. Weiss A. D. (1996). Role of social desirability in personality testing for personnel selection: the red herring. *J. Appl. Psychol.* 81 660–679. 10.1037/0021-9010.81.6.660

[B15] Paulhus D. L. (1984). Two-component models of socially desirable responding. *J. Pers. Soc. Psychol.* 46 598–609. 10.1037/0022-3514.46.3.598

[B16] Pauls C. A. Crost N. W. (2005). Cognitive ability and self-reported efficacy of self-presentation predict faking on personality measures. *J. Individ. Dif.* 26 194–206. 10.1027/1614-0001.26.4.194

[B17] Plato (2014). *The Republic*. BookRix.

[B18] Porter L. S. Philips C. Dickens S. Kiyak H. A. (2000). Social desirability in patients seeking surgical treatment for dentofacial disharmony: associations with psychological distress and motivation for treatment. *J. Clin. Psychol. Med. Settings* 7 99–106. 10.1023/A:1009539430402

[B19] Preiss M. Mačudová G. (2013). Dotazník žádoucího stylu odpovídání (BIDR-CZ). *Psychiatrie* 17 59–64.

[B20] Robins R. W. Beer J. S. (2001). Positive illusions about the self: short-term benefits and long-term cista. *J. Pers. Soc. Psychol.* 80 340–352. 10.1037/0022-3514.80.2.340 11220450

[B21] Rosse J. G. Stecher M. D. Miller J. L. Levin R. A. (1998). The impact of response distortion on preemployment personality testing and hiring decisions. *J. Appl. Psychol.* 83 634–644. 10.1037/0021-9010.83.4.634

[B22] Ruch F. L. Ruch W. W. (1967). The K factor as a (validity) supressor variable in predicting success in selling. *J. Appl. Psychol.* 51 201–204. 4382906 10.1037/h0024663

[B23] Viswesvaran C. Ones D. S. (1999). Meta-analysis of fakability estimates: implications for personality measurement. *Educ. Psychol. Meas.* 59 197–210. 10.1177/00131649921969802

[B24] Viswesvaran C. H. Ones D. S. Hough L. M. (2001). Do impression management scales in personality inventories predict managerial job performance ratings? *Int. J. Sel. Assess.* 9 277–289. 10.1111/1468-2389.00180

[B25] Vogt D. S. Colvin C. R. (2005). Assessment of accurate self-knowledge. *J. Pers. Assess.* 84 239–251. 10.1207/s15327752jpa8403_03 15907160

[B26] Vrcha P. (2006). Kvýběru Kandidátù na Funkci Soudce. Právní Rozhledy 23/2006.

[B27] Vůjtěch J. Holas J. Zeman P. (2002). *Profesiogram Soudce a Státního Zástupce. Profesiografická Studie Zaměřená na Výběr čekatelů a Výběrová Kriteria.* Praha: Institut pro kriminologii a sociální prevenci.

